# Supplementary material for: Comparing the number of outdoor sugar-sweetened beverage and caffeinated beverage advertisements near schools by school type and school-level economic advantage
Source: PLoS One. 2024 May 23;19(5):e0302048. doi: 10.1371/journal.pone.0302048 (PMC11115223; doi:10.1371/journal.pone.0302048)
Supplement: Attachment — Submitted filename: Response to Reviewers.docx [file pone.0302048.s002.docx]

Response to Reviewers

*Reviewer #1: Nice job responding to our comments. The only suggestion I have is to update your background - or at least this sentence: "As of 2014, nearly two thirds of children in the United States consumed at least one SSB per day." Since 2014 is now ten years ago it seems odd to include data so old. This is still a problem in 2024 so I request that you add at least one sentence that demonstrates this (i.e. data from 2019 at the earliest, but ideally from 2022 or '23 perhaps?)*

Thank you. We have revised this sentence to report the most recent data for the United States that were nationally representative, however, the most recent data were for 2018. If there is a specific paper that the reviewer is aware of that reports on the prevalence of sugar-sweetened beverage consumption for the US that is more recent we are happy to revise.

*Reviewer #2: Thank you for your close attention to the comments from reviewers, and for being transparent and upfront about the discovery of a coding error that altered your findings. I have no additional major comments. My one additional request is that you include a reference for the bootstrapping procedure used. Apart from that, I look forward to reading the final version of the manuscript.*

Thank you. We have added a reference for the bootstrapping procedure.
